# Mitochondrial ROS dyshomeostasis: a key driver of accelerated supraspinatus atrophy after rotator cuff injury

**DOI:** 10.3389/fphys.2026.1783596

**Published:** 2026-03-12

**Authors:** Erkai Pang, Yijin Zou, Kongye Lu, Jian Li, Xuxu Chen, Yu Zhu, Tao Wang, Linlin Shi, Hui Kang

**Affiliations:** 1 Department of Sports Medicine, Honghui Hospital, Xi’an Jiaotong University, Xi’an, Shaanxi, China; 2 Faculty of Science, National University of Singapore (NUS), Singapore, Singapore; 3 Department of Biosciences, University of Oslo, Oslo, Norway

**Keywords:** mitochondrial dysfunction, mitochondria-targeted antioxidants, muscle wasting pathways, oxidative stress, ROS, rotator cuff injury, supraspinatus atrophy

## Abstract

Rotator cuff injuries are common musculoskeletal disorders and are frequently accompanied by progressive supraspinatus muscle atrophy, which severely compromises functional recovery and surgical outcomes. Accumulating evidence indicates that mitochondrial reactive oxygen species (mtROS) dyshomeostasis is a central pathological driver of post-injury muscle degeneration. This review synthesizes current knowledge on the anatomical and histopathological changes following rotator cuff tears and focuses on the mechanisms governing mitochondrial ROS production, clearance, and dysregulation in the supraspinatus muscle. We highlight how excessive mtROS contribute to oxidative damage, mitochondrial dysfunction, impaired energy metabolism, and activation of key atrophy-related signaling pathways, including FOXO, NF-κB, MAPK, the ubiquitin-proteasome system, and the autophagy-lysosome pathway. Particular emphasis is placed on the unique biomechanical unloading, ischemic stress, and metabolic vulnerability of the supraspinatus following rotator cuff injury, which predispose this muscle to ROS-driven degeneration. Finally, we critically evaluate emerging therapeutic strategies targeting mtROS, including mitochondria-targeted antioxidants and conventional redox-modulating interventions, and discuss their translational potential and current limitations.

## 1 Introduction

Rotator cuff tears (RCTs) are a leading cause of shoulder pain and dysfunction, accounting for nearly 50% of shoulder-related disorders (Paolucci et al., 2023). They commonly arise from repetitive overhead activities or acute trauma and result in disruption of the rotator cuff’s force-coupling mechanism, leading to abnormal humeral head migration and impaired shoulder biomechanics (Thankam et al., 2016). The pathological spectrum ranges from tendinopathy to full-thickness tears, often accompanied by retraction and degeneration (Nayak et al., 2025). RCT prevalence increases sharply with age, from <1% in individuals aged 20–49 to over 36% in those ≥80 years (Bedi et al., 2024). Other risk factors include trauma, sex, and hand dominance. In addition to their clinical burden, RCTs also carry significant socioeconomic impact, with annual healthcare costs exceeding $34,000 per patient in the United States (Prasetia et al., 2023).

A major complication of chronic RCTs is supraspinatus muscle atrophy, primarily driven by mechanical unloading and neuromuscular inactivity (Hyatt and Powers, 2021). As a key dynamic stabilizer of the glenohumeral joint, the supraspinatus is particularly susceptible to progressive atrophy and fatty infiltration, both of which severely impair surgical repair outcomes (Prasetia et al., 2023). Although arthroscopic rotator cuff repair (ARCR) is widely employed, with reported success rates over 95%, its efficacy declines sharply in cases of advanced muscle degeneration (Longo et al., 2020). Over 270,000 ARCR procedures are performed annually in the U.S., and approximately 9,000 in the U.K. (Paolucci et al., 2023). The surgery typically involves tendon reattachment to the greater tuberosity using suture-anchor techniques (Zhang Y. et al., 2023). However, high retear rates persist postoperatively, ranging from 10% to 48.4%, and up to 94% in massive tears. Revision surgeries are technically challenging, associated with longer operative times, greater complication risks, and worse functional recovery (Eckers et al., 2023).

**FIGURE 1:**
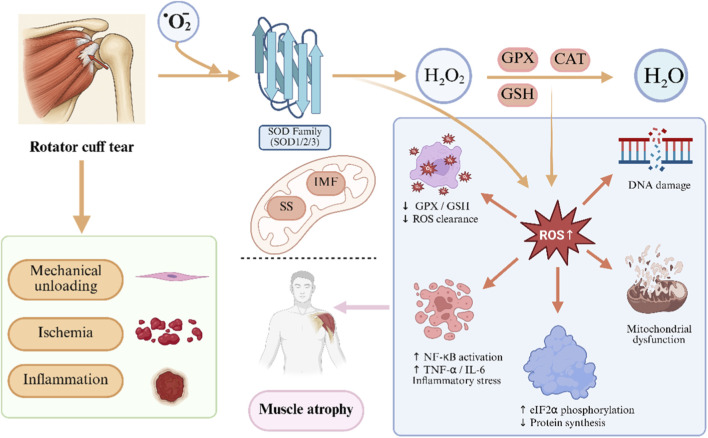
Mitochondrial ROS imbalance and pathological effects after rotator cuff tear Rotator cuff injury triggers ischemia, inflammation, and unloading, leading to excess ROS production-mainly superoxide from Complexes I and III. SOD enzymes convert superoxide to H_2_O_2_, but impaired antioxidant systems (e.g., GSH, GPX) allow H_2_O_2_to accumulate. Resulting ROS overload induces mitochondrial dysfunction, DNA damage, inflammatory cytokine release, NF-κB activation, and eIF2α phosphorylation, ultimately inhibiting protein synthesis and driving muscle atrophy. Subsarcolemmal mitochondria show early membrane damage; intermyofibrillar mitochondria are more prone to apoptosis.

Beyond mechanical degeneration, increasing evidence implicates mitochondrial dysfunction and oxidative stress in the pathogenesis of supraspinatus atrophy following RCTs (Lei et al., 2024). Prolonged tendon detachment and joint disuse elevate mitochondrial production of reactive oxygen species (ROS), which-at pathological levels-induce oxidative damage to proteins, lipids, and DNA (Zhao et al., 2023). ROS accumulation is exacerbated by mitochondrial membrane disruption, loss of membrane potential, and impaired ATP synthesis (Agrawal et al., 2026). Meanwhile, inflammatory cytokines and joint immobilization amplify oxidative injury, while antioxidant defenses such as superoxide dismutase (SOD), catalase, and glutathione peroxidase (GPx) become overwhelmed (Powers et al., 2012). In RCTs, this imbalance activates proteolytic pathways including the ubiquitin-proteasome system (UPS), autophagy, and caspase-3-mediated degradation. Moreover, ROS disrupt intracellular calcium homeostasis by inducing endoplasmic reticulum stress and activating calcium-dependent proteases such as calpains, further accelerating muscle protein degradation (Fukai and Ushio-Fukai, 2011). This mitochondrial ROS imbalance and its pathological effects are summarized in Figure 1.

Despite growing interest in oxidative stress and skeletal muscle atrophy, most existing reviews address ROS signaling in a generalized muscle context or focus primarily on tendon pathology after rotator cuff injury. A new review centered specifically on mitochondrial ROS (mtROS) dyshomeostasis within the supraspinatus muscle-integrating its unique biomechanical unloading, ischemic microenvironment, and post-injury metabolic stress-is still lacking. Moreover, how mtROS-driven redox imbalance links histopathological degeneration to downstream atrophy-related signaling pathways has not been comprehensively discussed in prior reviews.

In this review, we provide an mtROS-centered framework to bridge this gap by integrating anatomical and histopathological changes with mitochondrial dysfunction, redox dysregulation, and catabolic signaling in rotator cuff injury-induced supraspinatus atrophy. By emphasizing mitochondria-specific mechanisms and their therapeutic implications, this work aims to offer a focused mechanistic perspective and highlight potential translational targets for mitigating muscle degeneration after rotator cuff injury.

## 2 Methods

A comprehensive literature search was conducted to identify studies relevant to mitochondrial reactive oxygen species (mtROS), skeletal muscle atrophy, and supraspinatus degeneration following rotator cuff injury. The databases PubMed, Web of Science, and Scopus were searched for articles published up to 2025. Search terms included combinations of “mitochondrial ROS”, “oxidative stress”, “skeletal muscle atrophy”, “supraspinatus”, “rotator cuff injury”, “muscle unloading”, and “mitochondrial dysfunction”, using appropriate Boolean operators.

Studies were included if they investigated mtROS-related mechanisms in skeletal muscle, addressed muscle atrophy or disuse with relevance to mitochondrial function, or examined rotator cuff injury-associated supraspinatus pathology. Articles not related to skeletal muscle or mitochondrial biology, lacking relevance to rotator cuff injury or muscle atrophy, or consisting solely of commentaries or conference abstracts were excluded.

**FIGURE 2:**
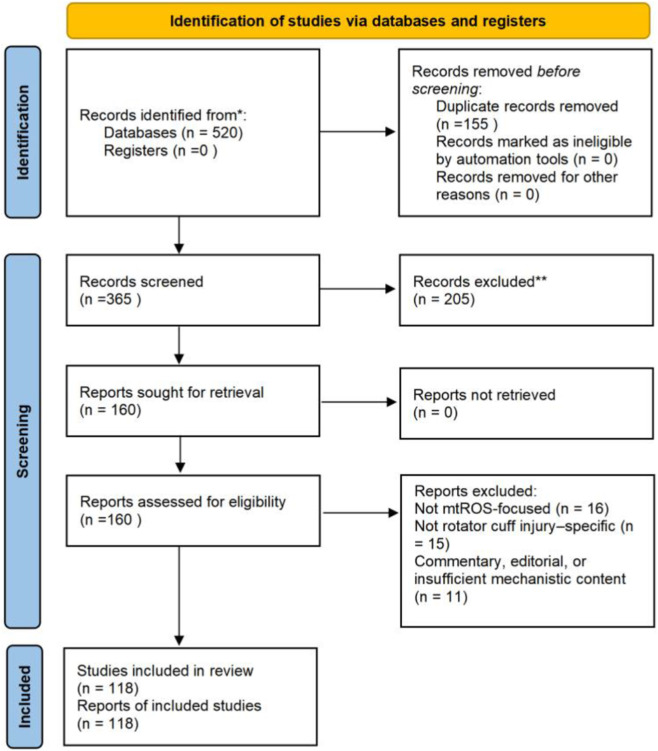
PRISMA 2020-style flow diagram of literature search and study selection. Records were identified through searches of PubMed, Web of Science, and Scopus databases up to 2025, followed by duplicate removal, title and abstract screening, and full-text eligibility assessment. The numbers of records excluded at each stage and the reasons for full-text exclusion are indicated.

All records were initially screened by title and abstract, followed by full-text assessment for eligibility. The overall process of literature identification, screening, eligibility assessment, and final inclusion is summarized in a PRISMA 2020-style flow diagram (Figure 2).

## 3 Pathophysiological mechanisms of supraspinatus atrophy after rotator cuff injury

### 3.1 Anatomy of the rotator cuff

The rotator cuff consists of four muscles-subscapularis, supraspinatus, infraspinatus, and teres minor-that originate from the scapula and insert onto the humeral tubercles. Functionally, they facilitate shoulder rotation and abduction while stabilizing the glenohumeral joint by centralizing the humeral head. Their tendons converge near the insertion sites, forming a cuff-like structure integrated with the joint capsule to support dynamic motion (Brand, 2008).

The supraspinatus and infraspinatus are innervated by the suprascapular nerve, the teres minor by the axillary nerve, and the subscapularis by the upper and lower subscapular nerves. Additional stabilizing structures include the rotator cuff-capsule complex, subacromial bursa, coracoacromial arch, and the long head of the biceps tendon. Together, these elements maintain shoulder stability and functional biomechanics (Hyatt and Powers, 2020).

### 3.2 Histological and imaging features of supraspinatus atrophy following rotator cuff injury

Chronic rotator cuff tears (RCTs) are characterized by tendon retraction, muscle atrophy, fatty infiltration, and fibrosis. Supraspinatus degeneration typically progresses through early, intermediate, and late stages-marked by inflammation, structural disruption, and irreversible degeneration, respectively. Early changes include reduced cross-sectional area (CSA) and fiber length; intermediate stages show fiber-type shifts, sarcomere disorganization, and fat/connective tissue accumulation; late stages present severe atrophy and impaired regenerative capacity. In murine models, acute inflammation peaks by day 5, with monocyte infiltration and upregulation of proinflammatory cytokines. Proteomic analyses reveal suppressed muscle metabolism, enhanced ECM remodeling, and time-dependent alterations in satellite cells and fibro-adipogenic progenitors (FAPs) (Gibbons et al., 2017).

Human biopsy studies echo these findings. In massive RCTs, muscle content is minimal (∼10%), with predominant fat and connective tissue. Over 90% of samples show fiber disorganization, adipose replacement, and macrophage infiltration (Frich et al., 2021). MRI, especially T1-weighted imaging, enables noninvasive assessment: greater tendon retraction correlates with reduced CSA (P < 0.001), fiber atrophy (P = 0.004), and lower muscle occupation ratio (r = −0.725) (Levin et al., 2023). When fat content exceeds 10%, diffuse infiltration becomes apparent. Medial-plane MRI slices offer improved atrophy evaluation but may underestimate severity under the Warner classification system.

Rotator cuff disruption not only produces structural degeneration but also alters shoulder biomechanics in a way that chronically unloads the supraspinatus. Electromyographic studies in patients with symptomatic rotator cuff tears demonstrate compensatory activation patterns characterized by increased activity of the biceps brachii and posterior deltoid, accompanied by reduced coordinated activation of the remaining rotator cuff muscles. This redistribution of muscular recruitment shifts functional demand away from the supraspinatus, leading to sustained contractile inactivity. Such unloading after tendon detachment differs from simple limb disuse, as it occurs in a context of altered tension, tendon retraction, inflammation, and impaired force transmission (Veen et al., 2021).

Importantly, muscle inactivity is metabolically active rather than neutral. Reduced contractile activity lowers ATP turnover and disturbs mitochondrial respiratory flux, increasing electron leakage from complexes I and III of the electron transport chain and promoting mitochondrial superoxide and hydrogen peroxide generation. In parallel, inactivity can enhance non-mitochondrial reactive oxygen species production through NADPH oxidases, disrupt calcium homeostasis, and impair mitochondrial quality-control mechanisms such as mitophagy. Diminished activation of endogenous antioxidant systems further compromises redox buffering capacity, allowing oxidative damage to accumulate. The supraspinatus may be particularly vulnerable to these processes because rotator cuff injury creates a unique microenvironment characterized by mechanical unloading, tendon discontinuity, local inflammation, and potential perfusion deficits (Zhou et al., 2024).

Together, compensatory biceps-dominant activation and chronic supraspinatus unloading provide a mechanistic link between rotator cuff injury and sustained mitochondrial reactive oxygen species elevation, thereby facilitating activation of proteolytic and atrophy-related signaling pathways that drive progressive muscle degeneration.

### 3.3 Molecular mechanisms of supraspinatus atrophy

Supraspinatus atrophy in chronic rotator cuff tears (RCTs) results from disrupted protein homeostasis, neuromuscular impairment, inflammation, mitochondrial dysfunction, oxidative stress, and circadian rhythm dysregulation (Krieger et al., 2017). Normally, the IGF-1/PI3K/Akt/mTOR pathway supports protein synthesis and inhibits catabolism via suppression of FoxO and E3 ligases such as MuRF1 and Atrogin-1 (Wan et al., 2022). Inflammatory or disuse conditions suppress this anabolic signaling and activate proteolytic systems including the ubiquitin-proteasome system (UPS), autophagy-lysosome pathway (ALP), calpains, and caspase-3 (Smuder et al., 2018).

Oxidative stress further amplifies proteolysis by increasing Beclin-1 and LC3-II expression and stimulating 20S proteasome activity, independent of ubiquitination. ROS also induce calcium dysregulation, activating calpains and caspase-3 to accelerate cytoskeletal breakdown (Ji et al., 2022). Neuromuscular instability-driven by impaired acetylcholine signaling and reduced neurotrophic factors (NGF, BDNF)-mimics denervation and promotes atrophy (Pascual-Fernández et al., 2020). Concurrently, TNF-α, IL-1β, and IL-6 activate NF-κB, JAK/STAT, and MAPK pathways, enhancing protease expression and suppressing myogenesis (He et al., 2025). Inflammatory stress also triggers HDAC4 activation, disrupting myogenic transcriptional programs via Dach2 and MYOG inhibition (Le et al., 2024).

**FIGURE 3:**
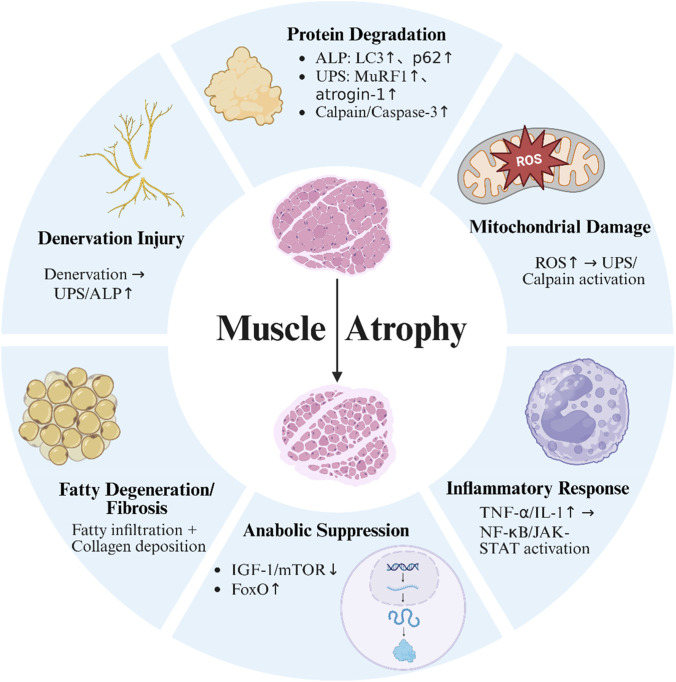
Key molecular mechanisms underlying skeletal muscle atrophy. This schematic summarizes six major contributors to muscle atrophy: (1) activation of proteolytic systems, including the autophagy-lysosome pathway, ubiquitin-proteasome system, calpains, and caspase-3; (2) mitochondrial dysfunction and ROS accumulation; (3) inflammation mediated by TNF-α and IL-1β; (4) inhibition of IGF-1/mTOR signaling and activation of FoxO transcription factors; (5) neuromuscular denervation; and (6) fat infiltration and fibrosis.

Mitochondrial dysfunction is central to chronic muscle loss, with supraspinatus fibers exhibiting reduced membrane potential, impaired ATP production, and increased ROS levels following RCTs (Hyatt et al., 2019). These changes promote oxidative damage, activate catabolic signaling, and create a vicious cycle of mitochondrial injury and calcium dysregulation. ROS further drive inflammation and tissue degeneration (Kim et al., 2023). Additionally, intrinsic factors such as genetic mutations (e.g., DMD, ALS) and circadian rhythm disruption contribute to muscle wasting. CLOCK and BMAL1 regulate muscle metabolism, mitochondrial dynamics, and antioxidant defense; their disruption compromises redox homeostasis and accelerates protein degradation (Yazdani et al., 2022). Figure 3 summarizes the key molecular mechanisms underlying muscle atrophy.

## 4 Mitochondrial ROS production, clearance, and dysregulation

### 4.1 Generation of mitochondrial ROS

Mitochondria are essential organelles responsible for ATP production via oxidative phosphorylation (OXPHOS), and they play central roles in calcium handling, apoptosis, biosynthetic metabolism, and redox regulation. In skeletal muscle-especially oxidative type I fibers-high mitochondrial density supports continuous aerobic activity. Reactive oxygen species (ROS), including superoxide anion (O_2_^−^·), hydrogen peroxide (H_2_O_2_), hydroxyl radical (·OH), and singlet oxygen (^1^O_2_), are generated as byproducts of mitochondrial respiration. While some ROS act as signaling molecules under physiological conditions, excess production disrupts redox homeostasis and damages proteins, lipids, and DNA (Hernansanz-Agustín and Enríquez, 2021).

Mitochondria are the primary source of intracellular ROS, primarily generated during electron transfer through complexes I-IV of the electron transport chain (ETC) (Michaelson et al., 2013). NADH and FADH_2_ donate electrons that reduce molecular oxygen to water, driving ATP synthesis at complex V (ATP synthase). However, approximately 1%–2% of oxygen undergoes incomplete reduction, especially at complexes I and III, leading to electron leakage and superoxide generation. This electron leak positions mitochondria as the dominant source of endogenous ROS in most aerobic cells (Lian et al., 2022). This figure illustrates the major intracellular sources of ROS, including the mitochondrial electron transport chain (see Supplementary Appendix Figure 1 in the attachment for detailed content).

### 4.2 Mitochondrial ROS scavenging systems

Excessive reactive oxygen species (ROS) accumulation leads to lipid peroxidation, mitochondrial membrane depolarization, cytochrome c release, caspase activation, and mitochondria-dependent apoptosis (Di Meo et al., 2019). Mitochondria generate superoxide (O_2_•^-^), rapidly converted to hydrogen peroxide (H_2_O_2_) by superoxide dismutases (SOD1 in cytosol/intermembrane space, SOD2 in the matrix, and SOD3 extracellularly) (Yang et al., 2020). While H_2_O_2_ acts as a physiological signaling molecule, its excess generates hydroxyl radicals (•OH) via Fenton chemistry, causing irreversible damage to proteins, lipids, and DNA. H_2_O_2_ also mediates redox signaling through Nrf2, AMPK/PGC-1α, and MAPK/ERK pathways, modulating antioxidant defense, metabolism, and cell survival (Chatzinikita et al., 2023).

**FIGURE 4:**
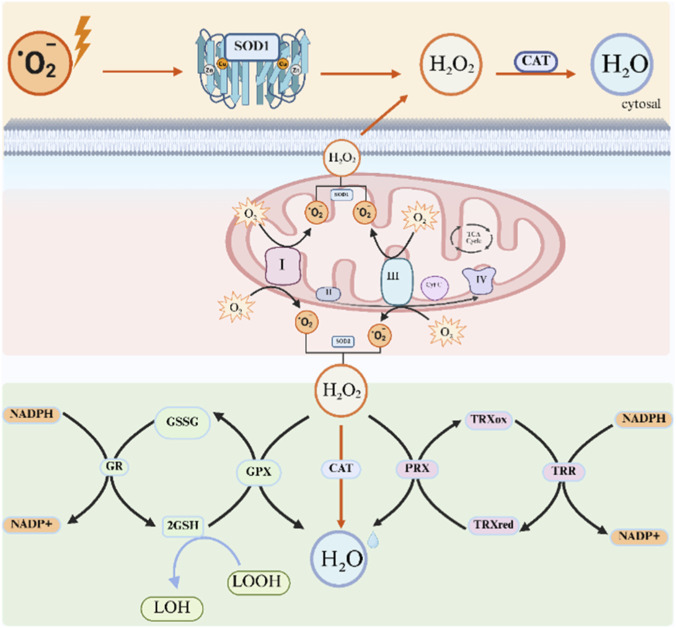
Mitochondrial antioxidant defense systems against ROS. This figure illustrates key mitochondrial enzymes that detoxify ROS. Superoxide (O_2_•^-^) produced at Complexes I and III is converted to hydrogen peroxide (H_2_O_2_) by SOD1 (intermembrane space) and SOD2 (matrix). H_2_O_2_ is then cleared via: (1) the GPX system (GPX1/4 uses GSH, regenerated by GSR with NADPH); (2) the Prx system (Prx3/5, regenerated by Trx/TrxR, also NADPH-dependent); and (3) the CAT system, which dismutates H_2_O_2_ into water and oxygen, mainly in peroxisomes and near mitochondria.

To neutralize ROS, mitochondria use enzymatic systems including glutathione peroxidases (GPX1/4), which reduce H_2_O_2_ and lipid peroxides using glutathione (GSH). GSH is regenerated from its oxidized form (GSSG) by glutathione reductase (GSR) with NADPH (Iannuzzo et al., 2024). Peroxiredoxins (Prx3/5) also scavenge H_2_O_2_, relying on thioredoxin (Trx) and thioredoxin reductase (TrxR) for regeneration. Catalase (CAT), mainly in peroxisomes but present in mitochondria of some tissues, decomposes H_2_O_2_ into water and oxygen rapidly, offering a transcriptionally regulated, redox-independent response (Robichaux et al., 2023). Together, these systems form a robust mitochondrial antioxidant defense critical for maintaining cellular redox balance. Figure 4 illustrates the key mitochondrial antioxidant defense systems against ROS.

### 4.3 Mechanisms and consequences of mitochondrial ROS imbalance following rotator cuff injury

Mitochondria are the primary source of reactive oxygen species (ROS) in skeletal muscle, and oxidative stress is significantly exacerbated after rotator cuff injury (Thankam et al., 2018). In the supraspinatus, chronic ischemia, mechanical unloading, and inflammation elevate mitochondrial ROS production, while antioxidant defenses-such as SODs, GPXs, and glutathione-are impaired, disrupting redox balance. Muscle mitochondria include subsarcolemmal (SS) and intermyofibrillar (IMF) subpopulations (Lui et al., 2022). SS mitochondria, located beneath the sarcolemma, are more susceptible to early oxidative damage, whereas IMF mitochondria, with higher oxidative capacity, become increasingly sensitive to apoptotic signals during injury (Ji and Yeo, 2019).

While physiological ROS levels regulate adaptive pathways via Nrf2 and AMPK/PGC-1α signaling, excessive ROS become deleterious (Zhang et al., 2019). They promote lipid, protein, and DNA oxidation, forming cytotoxic products such as malondialdehyde (MDA) and 4-hydroxynonenal (4-HNE), which compromise mitochondrial DNA and disrupt electron transport, especially at complexes I and III (Wang et al., 2024).

In addition, ROS inhibit protein synthesis by inducing phosphorylation of eIF2α, blocking translation initiation (Seo et al., 2022). These alterations create a vicious cycle of mitochondrial dysfunction, oxidative injury, and myofiber apoptosis, ultimately leading to irreversible supraspinatus atrophy and impaired regeneration after rotator cuff tears.

## 5 Mitochondrial ROS imbalance in the molecular pathogenesis of supraspinatus muscle atrophy

### 5.1 Oxidative stress-induced damage triggered by ROS imbalance

#### 5.1.1 Oxidative modification of proteins, lipids, and DNA

Reactive oxygen species (ROS) induce oxidative damage to skeletal muscle macromolecules, disrupting structural integrity and biological function. Proteins are particularly susceptible, with ROS oxidizing amino acid side chains-especially cysteine and methionine-leading to peptide cleavage and aberrant cross-linking. Oxidation of cysteine’s thiol group yields sulfenic (R-SOH), sulfinic (R-SO_2_H), and sulfonic (R-SO_3_H) acids, while carbonylation of arginine, lysine, and threonine, as well as tyrosine nitration, generates protein carbonyls (PCs), established oxidative stress markers (Panella et al., 2025). In parallel, reversible oxidative post-translational modifications (Ox-PTMs) such as S-glutathionylation (PSSG), S-nitrosylation (SNO), and disulfide bond formation fine-tune redox signaling but become maladaptive under persistent stress (Zhang T. et al., 2021). In muscle, excess ROS increase PSSG and SNO levels, impairing enzyme activity. For instance, mitochondrial thymidine kinase 2 (TK2) is glutathionylated upon H_2_O_2_ exposure, leading to its inactivation and proteasomal degradation.

Lipids, especially polyunsaturated fatty acids (PUFAs) in membrane phospholipids, are prime targets for ROS-induced peroxidation (Gentile et al., 2021). The process proceeds via initiation (hydrogen abstraction), propagation (lipid peroxyl radical formation), and termination. Resulting lipid hydroperoxides (LOOHs) degrade into reactive aldehydes such as malondialdehyde (MDA) and 4-hydroxy-2-nonenal (4-HNE). These byproducts can diffuse to modify proteins and nucleic acids through covalent adduction (Eskelinen et al., 2022). Notably, 4-HNE acts as a signaling molecule that promotes muscle atrophy by activating FoxO transcription factors and suppressing Wnt/β-catenin signaling (Almeida et al., 2009).

ROS also damage nucleic acids, particularly guanine bases, resulting in eight-oxoguanine formation, DNA strand breaks, and telomere shortening (Bloemberg and Quadrilatero, 2019). Skeletal muscle’s limited DNA repair capacity makes it particularly vulnerable. ROS-induced DNA lesions activate the p53 pathway, promoting Bax expression, mitochondrial cytochrome c release, apoptosome assembly, and caspase-9/3 activation, culminating in apoptosis (Zhou et al., 2025).

Following rotator cuff injury, supraspinatus muscle exhibits prominent oxidative damage. ROS-mediated DNA injury compromises satellite cell regenerative capacity, while oxidized macromolecules accumulate within atrophic fibers (Ionescu et al., 2025). These biomolecular insults trigger degradation cascades and inflammation via damage-associated molecular patterns (DAMPs), establishing a self-perpetuating loop of oxidative stress and muscle degeneration. Mitochondrial ROS imbalance is now recognized not only as a key driver of muscle atrophy and apoptosis, but also as a contributor to broader pathologies including aging, carcinogenesis, male infertility, and colorectal cancer (Nakazzi et al., 2025).

#### 5.1.2 Oxidative stress-induced structural and functional impairment of muscle fibers

Oxidative stress (OS) is a critical mediator of structural and functional deterioration in skeletal muscle. Owing to their high oxygen consumption, skeletal muscle fibers are inherently prone to ROS generation during contraction and metabolism (Kann et al., 2022). When ROS levels exceed endogenous antioxidant capacity, oxidative modifications to proteins, lipids, and DNA ensue-compromising membrane integrity, organelle function, and cellular homeostasis. Additionally, ROS impair calcium signaling by oxidizing key ion channels such as ryanodine receptor 1 (RyR1) and dihydropyridine receptor (DHPR), reducing calcium sensitivity and disrupting excitation-contraction coupling.

Elevated ROS also activate the NF-κB pathway by depleting glutathione (GSH), downregulating myogenic transcription factors (MyoD, MyoG), and upregulating the transcriptional repressor Yin Yang 1 (YY1), thereby impairing myogenic differentiation (Xiang et al., 2022). Furthermore, ROS suppress p21 expression and increase apoptosis in myogenic progenitor cells during early myogenesis. Chronic oxidative stress induces premature senescence of muscle stem cells (MuSCs), diminishes their self-renewal capacity, and downregulates the SIRT1/Nrf2 axis, weakening antioxidant and DNA repair systems (Canfora et al., 2022). Notably, OS displays fiber-type specificity: in slow-twitch (soleus) muscle, SOD upregulation is accompanied by reductions in peroxiredoxin 6 (PRDX6) and carbonic anhydrase III (CAH III), exacerbating H_2_O_2_ accumulation and oxidative damage; fast-twitch (gastrocnemius) muscle better maintains redox balance. Trolox supplementation has been shown to mitigate OS in soleus fibers, underscoring ROS as active contributors to muscle atrophy (Wu et al., 2024).

Importantly, ROS are not inherently deleterious. At physiological levels, they serve as signaling molecules that promote exercise-induced adaptations, such as mitochondrial biogenesis via PGC-1α and upregulation of intrinsic antioxidant systems. However, persistent ROS overproduction overrides adaptive pathways and transforms redox signals into pathological triggers-driving fiber degradation, contractile dysfunction, and progressive muscle degeneration (Skinner et al., 2021).

### 5.2 ROS-mediated mitochondrial dysfunction and vicious cycle formation

#### 5.2.1 ROS-induced mitochondrial membrane damage and functional collapse

Excessive mitochondrial ROS are central mediators of membrane disruption and bioenergetic failure. Mitochondrial DNA (mtDNA), which encodes 13 essential subunits of the electron transport chain (ETC), is particularly susceptible to oxidative stress, resulting in replication errors, point mutations, and large-scale deletions (Kim and Kim, 2018). Loss of up to 25%–80% of the mtDNA genome compromises the assembly and function of, ETC., complexes, attenuates proton pumping, and leads to dissipation of mitochondrial membrane potential (ΔΨm) (Robichaux et al., 2023). The collapse of ΔΨm not only reflects impaired electron transport and ATP synthesis but also serves as a molecular trigger for mitochondrial dysfunction and downstream apoptotic signaling (Liu et al., 2020).

ROS further promote the pathological opening of the mitochondrial permeability transition pore (mPTP), leading to ΔΨm collapse, matrix swelling, inner membrane rupture, and the release of pro-apoptotic factors such as cytochrome c. This initiates caspase activation and myonuclear apoptosis (Bellanti et al., 2021). The mPTP is regulated by ROS and calcium flux, involving components such as cyclophilin D, adenine nucleotide translocator (ANT), and Bax/Bak. ROS enhance Bax/Bak oligomerization, increasing outer membrane permeability and enabling cytosolic leakage of mtDNA. This extracellular mtDNA acts as a danger-associated molecular pattern (DAMP), triggering inflammation and PARP-mediated cell death (Basse et al., 2021).

In addition, mitochondria form functional contact sites with the sarcoplasmic reticulum (SR) at mitochondria-associated membranes (MAMs), where Ca^2+^ is transferred through the IP_3_R-Grp75-VDAC1 complex. Under oxidative stress, excessive Ca^2+^ influx into mitochondria exacerbates mPTP activation and ΔΨm loss (Annesley and Fisher, 2019). Simultaneously, impaired calcium uptake due to dysfunctional mitochondrial calcium uniporter (MCU) hinders ΔΨm restoration and calcium buffering, further amplifying mitochondrial stress (Chen et al., 2023). Collectively, ROS-induced mtDNA damage, ΔΨm dissipation, mPTP activation, and Ca^2+^ dysregulation converge to drive mitochondrial collapse, ATP depletion, and skeletal myocyte apoptosis or necrosis-ultimately contributing to the pathogenesis of muscle atrophy (Chen et al., 2023).

#### 5.2.2 Mitochondrial dysfunction exacerbates ROS production: a vicious cycle

Mitochondrial dysfunction both amplifies ROS generation and impairs antioxidant defense, establishing a self-perpetuating cycle of oxidative injury and organelle deterioration (Chen T-H. et al., 2022). Mitochondrial dynamics, orchestrated by fusion proteins (mitofusin-1/2 [Mfn1/2] and optic atrophy 1 [OPA1]) and fission regulators such as dynamin-related protein 1 (Drp1), are essential for maintaining mitochondrial morphology, network integrity, and bioenergetic capacity (Yazdani et al., 2023). Under oxidative stress, this balance is disrupted-fusion is suppressed while fission is enhanced-resulting in mitochondrial fragmentation, cristae disorganization, and reduced efficiency of the electron transport chain (ETC). In models of cachexia and chronic muscle atrophy, expression of the fission-related protein Fis1 is upregulated, whereas Mfn1/2 levels are decreased, implicating ROS in both transcriptional and post-translational regulation of mitochondrial dynamics (Powers et al., 2016).

Mitophagy serves as a key quality control mechanism that selectively eliminates dysfunctional mitochondria, thereby limiting ROS accumulation and preserving metabolic homeostasis (Calvani et al., 2013). Moderate ROS levels promote mitophagy via the hypoxia-inducible factor-1α (HIF-1α)/BCL2-interacting protein 3 (BNIP3)/Beclin-1 axis. However, sustained ROS overload skews this protective mechanism toward dysfunction. Excessive mitophagy can deplete mitochondrial reserves and disrupt ATP production, while insufficient or inhibited mitophagy allows damaged mitochondria to persist, exacerbating oxidative stress and cellular injury (Ji et al., 2022).

In chronic ischemic conditions-such as rotator cuff tears-HIF-1α/BNIP3 signaling is disrupted, leading to impaired mitophagy homeostasis and persistent mitochondrial stress. These maladaptive changes establish a pathological feedback loop: ROS accumulation triggers mitochondrial fragmentation and mitophagy dysregulation, which in turn promotes further ROS production (Ji et al., 2022). This vicious cycle exacerbates supraspinatus muscle degeneration and contributes to the progression of muscle atrophy (Park et al., 2021).

### 5.3 ROS-activated signaling pathways in muscle atrophy

Following rotator cuff injury, the supraspinatus undergoes chronic functional unloading due to tendon discontinuity and compensatory recruitment of adjacent muscles. Unlike generalized limb disuse, this unloading occurs in a mechanically altered and inflammatory microenvironment characterized by tendon retraction, impaired force transmission, and local hypoperfusion. Reduced contractile activity decreases ATP turnover and slows oxidative phosphorylation flux, promoting partial reduction of electron transport chain components and enhanced electron leakage from complexes I and III. These electrons react with molecular oxygen to generate superoxide and subsequently hydrogen peroxide. Sustained unloading is further associated with impaired antioxidant defense, including diminished Nrf2-mediated transcription and reduced SOD2 activity, thereby limiting mitochondrial ROS detoxification. In parallel, altered mechanotransduction and inflammatory signaling may activate NADPH oxidase isoforms, providing additional non-mitochondrial ROS sources. Functional inactivity also disrupts calcium homeostasis; cytosolic calcium instability drives mitochondrial calcium overload, destabilizes membrane potential, increases permeability transition pore opening, and amplifies ROS generation. Impaired mitophagy permits accumulation of dysfunctional mitochondria, establishing a feed-forward cycle of oxidative stress within the supraspinatus.

**FIGURE 5:**
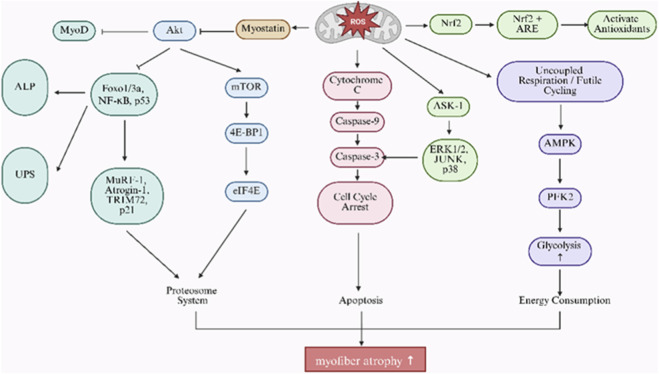
ROS-driven signaling pathways in skeletal muscle atrophy. Elevated ROS activate multiple catabolic cascades that contribute to muscle wasting. These include mitochondrial apoptosis (via cytochrome c release and caspase activation), inhibition of protein synthesis (through Akt-mTOR suppression), and activation of proteolytic systems (FoxO, NF-κB, UPS, ALP). ROS further modulate MAPK signaling (ERK, JNK, p38), weaken antioxidant defenses (via Nrf2 suppression), and disrupt energy homeostasis (through AMPK and PI3K pathways), collectively accelerating myofiber degeneration.

In this setting, excessive ROS act as active signaling mediators rather than passive metabolic byproducts. Elevated ROS activate FoxO-, NF-κB-, and MAPK-dependent catabolic pathways, stimulate ubiquitin–proteasome and autophagy–lysosome systems, and suppress PI3K/Akt/mTOR-mediated anabolic signaling. Concurrent disruption of the AMPK–Nrf2 axis further weakens antioxidant capacity and exacerbates mitochondrial dysfunction. As summarized in Figure 5, these interconnected ROS-driven mechanisms integrate mitochondrial impairment with proteolytic signaling cascades and provide a mechanistic explanation for progressive supraspinatus atrophy following rotator cuff injury.

#### 5.3.1 ROS-mediated inhibition of the AMPK pathway and antioxidant defense

AMP-activated protein kinase (AMPK) is a central regulator of cellular energy sensing and redox homeostasis in skeletal muscle (Ren et al., 2021). In muscle fibers, AMPK is predominantly composed of α2 and β2 subunits and is activated by Thr172 phosphorylation mediated by upstream kinases including LKB1, CaMKKβ, and TAK1 (Garcia and Shaw, 2017). Physiological levels of ROS can transiently activate AMPK through H_2_O_2_-mediated stimulation of LKB1 or Ca^2+^-dependent CaMKKβ signaling, as well as through redox-sensitive modifications such as S-glutathionylation (Guan et al., 2025).

Activated AMPK promotes antioxidant defense by phosphorylating Nrf2, facilitating its nuclear translocation and transcriptional induction of antioxidant enzymes including superoxide dismutase (SOD), catalase, and glutathione peroxidase (GPx). This AMPK-Nrf2 axis is critical for maintaining redox balance and mitochondrial integrity in skeletal muscle (Wang et al., 2022).

**FIGURE 6:**
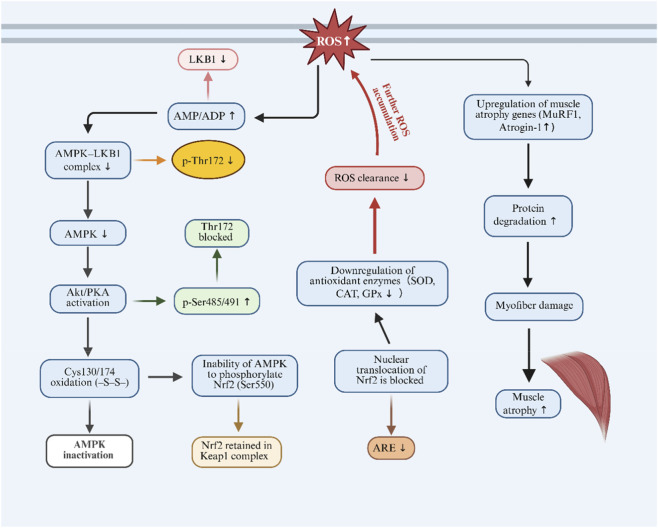
Disruption of the ROS-AMPK-Nrf2 axis in muscle atrophy. Excessive ROS impairs AMPK activation by disrupting LKB1 signaling, increasing AMP/ADP levels, promoting inhibitory Ser485/491 phosphorylation, and causing irreversible oxidation at key cysteine residues. Inactive AMPK fails to phosphorylate Nrf2 at Ser550, blocking its release from Keap1 and nuclear translocation. As a result, antioxidant enzyme expression (SOD, CAT, GPx) is reduced, ROS clearance is impaired, and oxidative stress intensifies. This dysregulation enhances atrogene expression (MuRF1, Atrogin-1), protein degradation, and myofiber loss, driving muscle atrophy.

In contrast, sustained or excessive ROS accumulation suppresses AMPK activity by destabilizing the AMPK-LKB1 complex, inducing inhibitory Ser485/491 phosphorylation via Akt/PKA signaling, and causing irreversible oxidative modifications of key cysteine residues (Yan et al., 2022). Concurrent impairment of Nrf2 signaling reduces antioxidant capacity, establishing a feed-forward loop characterized by progressive ROS accumulation, mitochondrial dysfunction, and muscle fiber degeneration. Disruption of the ROS-AMPK-Nrf2 axis has been implicated in muscle atrophy associated with disuse, ischemia-reperfusion injury, and metabolic stress (Coughlan et al., 2016). These mechanisms are summarized in Figure 6.

#### 5.3.2 Activation of NF-κB by ROS promotes inflammation and protein degradation

Nuclear factor-κB (NF-κB) is a key redox-sensitive transcription factor that regulates inflammation, cell survival, and protein catabolism in skeletal muscle (Roy et al., 2018). Under resting conditions, NF-κB dimers are retained in the cytoplasm by inhibitory proteins such as IκBα. Pathological stimuli-including inflammatory cytokines and oxidative stress-trigger IKK-mediated IκBα degradation, enabling NF-κB nuclear translocation and transcription of pro-inflammatory cytokines and atrophy-related genes (Scalabrin et al., 2020).

Reactive oxygen species (ROS), particularly hydrogen peroxide (H_2_O_2_), strongly potentiate NF-κB activation by stimulating redox-sensitive upstream kinases such as TAK1 and inhibiting phosphatases including PP2A, thereby accelerating IκBα degradation and nuclear signaling (Vainshtein and Sandri, 2020) (Gorza et al., 2021). ROS also enhance receptor-mediated signaling through TNFR and TLR4 and directly modify NF-κB subunits via cysteine oxidation, altering DNA-binding activity and transcriptional output (Zhang H. et al., 2023).

**FIGURE 7:**
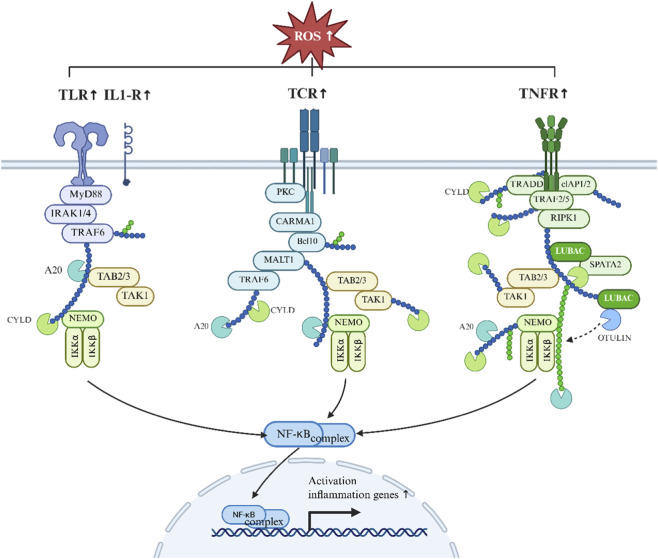
ROS-activated NF-κB signaling in muscle atrophy. ROS upregulate membrane receptors such as TLRs, TCRs, and TNFRs, triggering TRAF-mediated activation of downstream kinases (TAK1, TAB2/3, NEMO). This leads to IκBα phosphorylation and degradation by the IKK complex, releasing RelA:p50 for nuclear translocation. NF-κB then induces pro-inflammatory cytokines (TNF-α, IL-6), anti-apoptotic proteins, and proteolytic factors, contributing to inflammation and muscle protein breakdown.

While transient NF-κB activation contributes to adaptive inflammatory responses, sustained ROS accumulation maintains chronic NF-κB signaling, promoting muscle protein degradation, metabolic dysfunction, and inflammatory remodeling (Canfora et al., 2022). In addition, NF-κB exhibits extensive cross-talk with other redox-responsive transcription factors, including Nrf2, STAT3, HIF-1α, AP-1, and FoxO, integrating oxidative stress with inflammatory and catabolic signaling (Surai et al., 2021). One important downstream target is heme oxygenase-1 (HO-1), linking NF-κB activity to redox adaptation and antioxidant defense (Zhang M-H. et al., 2021). These ROS-driven NF-κB signaling events in muscle atrophy are summarized in Figure 7.

#### 5.3.3 ROS-mediated activation of FoxO and initiation of protein degradation programs

The forkhead box O (FoxO) transcription factors, particularly FoxO1 and FoxO3 in skeletal muscle, play a central role in regulating proteolysis by activating both the ubiquitin-proteasome system (UPS) and autophagy-lysosome system (ALS) (Oyabu et al., 2022). FoxOs transcriptionally induce E3 ligases like Atrogin-1/MAFbx and MuRF1, as well as autophagy genes including BNIP3, LC3, and Atg12. Under normal conditions, FoxO activity is inhibited by the IGF-1/PI3K/Akt pathway via Akt-mediated phosphorylation, which retains FoxOs in the cytoplasm through 14-3-3 protein binding (Kang et al., 2017). In catabolic conditions such as fasting or denervation, this inhibition is relieved, allowing nuclear translocation of FoxOs and activation of muscle atrophy programs (Lim et al., 2025).

Besides classical targets, FoxOs also regulate noncanonical E3 ligases such as MUSA1, FBXO31, SMART (FBXO21), and Itch, with FoxO3 as the dominant factor (Li et al., 2022). *In vivo* studies show that muscle-specific triple knockout of FoxO1/3/4 abolishes the induction of 29 atrogenes-including E3 ligases, autophagy mediators, deubiquitinases (e.g., USP14), and proteasome subunits (e.g., Psmd11)-under catabolic stimuli, preserving muscle mass and strength (Sanchez et al., 2014).

FoxO activity is finely regulated by cofactors such as HDAC6, PGC-1α, and GADD45α, and by miRNAs including miR-182, miR-486, and miR-23. Under oxidative stress, ROS act as major metabolic signals that enhance FoxO activation through multiple pathways: suppression of PI3K/Akt, activation of AMPK, JNK, and p38 MAPK, and deacetylation via SIRT1 and HDACs (Peris-Moreno et al., 2021). ROS also promote FoxO stability through oxidative post-translational modifications like S-nitrosylation and 4-HNE adducts (Zhang H. et al., 2023).

**FIGURE 8:**
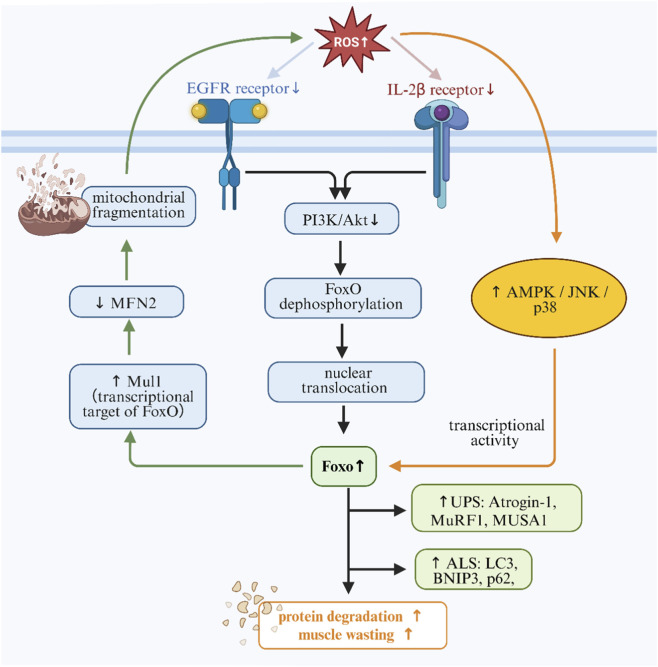
ROS-mediated activation of FoxO signaling in muscle protein degradation. Excess ROS suppress the PI3K/Akt pathway and activate AMPK, JNK, and p38 MAPK, facilitating FoxO dephosphorylation and nuclear translocation. Nuclear FoxO drives transcription of atrogenes such as Atrogin-1, MuRF1, and LC3, promoting proteolysis via UPS and ALS, leading to mitochondrial damage and muscle atrophy.

These mechanisms enhance FoxO-driven expression of both classical and noncanonical E3 ligases, robustly activating UPS (Chen K. et al., 2022). FoxO3 further induces autophagy via LC3, BNIP3, and p62, and upregulates Mul1, which degrades MFN2 and promotes mitochondrial fragmentation, forming a vicious cycle: “ROS → FoxO → mitochondrial damage → more ROS”. Additionally, FoxO coordinates autophagosome trafficking via HDAC6 and interacts with PGC-1α, TXN1, and GADD45α to integrate metabolic and antioxidant responses (Powers et al., 2016). ROS-induced caspase-3 and calpains also degrade cytoskeletal proteins, promoting UPS substrate supply. In FoxO1/3/4 knockout mice, ubiquitination and autophagy flux are nearly abolished, confirming the pivotal role of ROS-FoxO signaling in muscle wasting (Canovas and Nebreda, 2021). Figure 8 shows the ROS-mediated activation of FoxO signaling in muscle protein degradation.

#### 5.3.4 ROS activation of the MAPK pathway inhibits muscle protein synthesis

The mitogen-activated protein kinase (MAPK) family consists of evolutionarily conserved serine/threonine kinases that relay extracellular signals to intracellular targets, regulating proliferation, differentiation, apoptosis, and metabolism. In mammals, the four major MAPK cascades-ERK1/2, JNK, p38 MAPK, and ERK5-have distinct roles (Yuasa et al., 2018). ERK1/2 is typically activated by mitogens and supports cell growth and survival, while JNK and p38 MAPK are stress-activated protein kinases (SAPKs), triggered by oxidative stress, cytokines, or DNA damage. Accumulated ROS under pathological conditions-such as inflammation, ischemia, or denervation-activate upstream kinases (ASK1, MKK3/6, MKK4/7), leading to p38 and JNK phosphorylation. Notably, p38 MAPK is highly sensitive to oxidative signals, with low-dose H_2_O_2_ sufficient to induce its rapid activation in muscle cells, preceding activation of catabolic regulators like FoxO and NF-κB (Changchien et al., 2019).

Once activated, p38 MAPK and JNK promote transcription of muscle atrophy-related genes. p38 upregulates E3 ligases (Atrogin-1, MuRF1, Nedd4) and autophagy genes (Atg7), while JNK phosphorylates c-Jun and FoxO to enhance their pro-atrophic activity (Changchien et al., 2019). Meanwhile, ERK contributes by inducing early growth response genes (Egr1/2) and downstream effectors like RSK and MSK. Collectively, MAPKs activate both the ubiquitin-proteasome system (UPS) and the autophagy-lysosome system (ALS), accelerating sarcomeric protein and organelle degradation (Kim et al., 2015). Additionally, MAPK signaling suppresses the Akt-mTORC1 axis, reducing phosphorylation of p70S6K and 4EBP1, thus impairing translation initiation and ribosomal biogenesis. Prolonged MAPK activity also activates mitochondrial apoptotic pathways by phosphorylating Bcl-2 family proteins (e.g., Bcl-2, BAD, Bim), leading to caspase-dependent myofiber loss (Vainshtein and Sandri, 2020).

**FIGURE 9:**
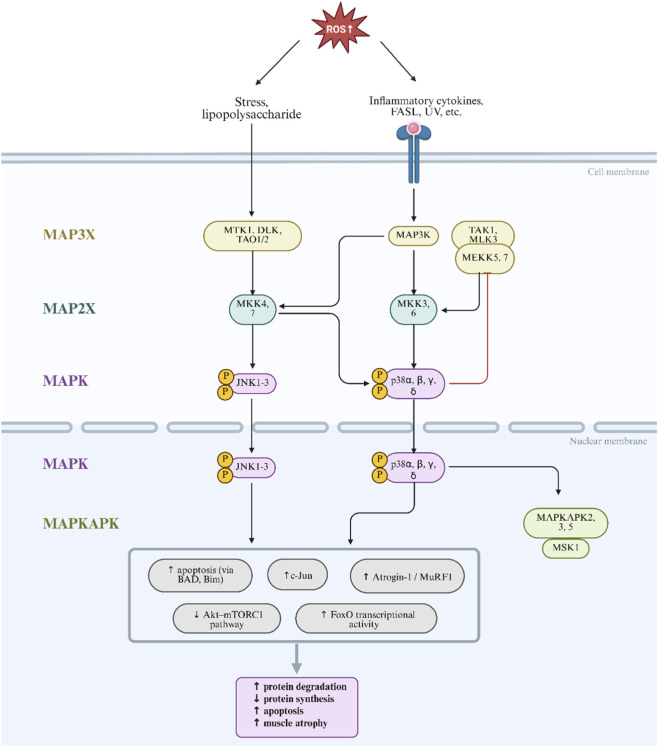
ROS-activated MAPK pathway promotes protein degradation and suppresses synthesis. Oxidative stress activates JNK and p38 MAPK signaling, inducing transcription factors such as c-Jun and FoxO. This upregulates atrogenes (MuRF1, Atrogin-1) and triggers apoptotic pathways. Concurrently, p38 inhibits the Akt–mTORC1 axis, suppressing translation initiation and protein synthesis, thereby accelerating muscle atrophy.

*In vivo* studies confirm the critical role of p38 MAPK in oxidative muscle catabolism. Pharmacological blockade with SB202190 mitigates H_2_O_2_-induced expression of Atrogin-1 and Atg7, preserving myotube morphology and attenuating atrophy. Interestingly, p38 exhibits context-dependent effects: under physiological conditions (e.g., exercise), p38α promotes mitochondrial biogenesis and fiber-type remodeling (Vainshtein and Sandri, 2020). Conversely, in pathological states like cancer cachexia, systemic inflammation, or renal failure, p38α initiates catabolic and apoptotic signaling cascades. A key downstream mediator is CaMK2B, which facilitates denervation-induced atrophy. Inhibition of CaMK2B via genetic or pharmacological approaches has been shown to reduce muscle loss in experimental models (Haberecht-Müller et al., 2021). Figure 9 depicts how the ROS-activated MAPK pathway promotes protein degradation and suppresses synthesis.

#### 5.3.5 ROS promotes UPS-mediated acceleration of protein degradation

The ubiquitin-proteasome system (UPS) is the primary protein degradation machinery in eukaryotic cells, responsible for removing misfolded, damaged, or excess proteins to maintain proteostasis and regulate key cellular processes including cell cycle and immune signaling (Peris-Moreno et al., 2021). UPS-mediated degradation involves a cascade of E1 (activating), E2 (conjugating), and E3 (ligating) enzymes that attach ubiquitin chains-typically linked via lysine-48-to target proteins for recognition by the 26S proteasome. This proteasome, comprising a 20S catalytic core and 19S regulatory subunits, possesses chymotrypsin-, trypsin-, and caspase-like activities (Peris-Moreno et al., 2021). In skeletal muscle atrophy, UPS selectively degrades sarcomeric proteins such as myosin heavy chain and MyBP-C. However, these large contractile proteins must first be disassembled by calcium-dependent proteases (e.g., calpains, caspases) to expose ubiquitin-recognizable sites. Glycogen synthase kinase-3β (GSK-3β), for instance, phosphorylates desmin to facilitate its cleavage and degradation Anon, 2018.

ROS indirectly accelerate UPS-mediated proteolysis by increasing intracellular calcium levels, activating calpains, and promoting cytoskeletal disassembly. Among the E3 ligases, MuRF1 and Atrogin-1/MAFbx are canonical “atrogenes” strongly upregulated during catabolic stress (e.g., denervation, fasting, glucocorticoid exposure, cancer cachexia), and are indispensable for UPS-mediated muscle degradation. MuRF1 primarily targets sarcomeric proteins and forms part of a Cullin4A-DDB1-DCAF8 E3 ligase complex, while Atrogin-1 preferentially degrades regulatory factors such as MyoD1 and eIF3-f. Knockout of either gene significantly reduces muscle wasting. Another key E3 ligase, TRIM32, contributes to cytoskeletal remodeling by promoting desmin degradation and modulating the PI3K/Akt/FOXO pathway. Mutations in TRIM32 are linked to LGMD2H and its upregulation is observed in Duchenne and Becker muscular dystrophies (Borges et al., 2021).

UPS activity is transcriptionally regulated by multiple factors. NRF1 (NFE2L1), a proteasome activity sensor localized to the endoplasmic reticulum, is normally degraded by ERAD. Under proteotoxic or oxidative stress, NRF1 is cleaved by DDI2 and translocated to the nucleus to activate genes encoding proteasome subunits, establishing a feedback response. NRF1 expression is upregulated in denervation-induced muscle atrophy (Borges et al., 2021). NRF2 also enhances proteasome function but is primarily involved in redox regulation. Other regulators include NRF3 (via NFE2L1 mRNA), FoxO, STAT3, and NF-Y. Additionally, PAX4 facilitates the late-stage UPS process by upregulating p97/VCP, which extracts ubiquitinated proteins from aggregates for proteasomal degradation. PAX4 inhibition delays myofibril breakdown, highlighting its role in advanced atrophy progression. Supplementary Appendix Figure 2. ROS accelerates skeletal muscle protein degradation via activation of the UPS (see Supplementary Appendix Figure 2 in the attachment for detailed content).

#### 5.3.6 ROS enhances autophagy-lysosomal system-mediated muscle structural disruption

The autophagy-lysosomal pathway (ALP) is an essential intracellular degradation system that maintains proteostasis by eliminating dysfunctional organelles and aggregated proteins. It proceeds via sequential steps: phagophore formation, autophagosome maturation, lysosomal fusion, and cargo degradation in autolysosomes. Under oxidative stress, ALP is activated to mitigate ROS-induced damage. Conversely, defective ALP exacerbates mitochondrial dysfunction and protein aggregation, creating a feed-forward loop that amplifies ROS levels and cytotoxicity (Tang et al., 2025).

Recent studies have identified transcription factor EB (TFEB) as a central regulator of lysosomal biogenesis and autophagy. Its nuclear translocation is modulated by redox status, particularly via ROS accumulation resulting from thioredoxin reductase (TrxR1/2) suppression (Yang et al., 2025). Activation of the ROS-p53-SESN2-TFEB/TFE3 axis promotes autophagic flux and lysosomal gene expression independent of nutrient signals. In cancer cells, TrxR1/2 inhibition by Hdy-7 induces cytotoxic autophagy through elevated ROS and TFEB activation, which can be reversed by antioxidants or p53 knockdown, illustrating the redox sensitivity of this pathway.

In skeletal muscle, ALP works in concert with UPS to degrade structural proteins under stress conditions such as disuse, hypoxia, or nutrient deprivation. These catabolic states increase ROS production, triggering both UPS and ALP activation. Human bone marrow mesenchymal stem cell-derived extracellular vesicles (hBMSC-EVs) have shown promise in counteracting ROS-induced ALP overactivation (Chang et al., 2025). *In vitro* and *in vivo* studies demonstrate that hBMSC-EVs reduce ROS, enhance antioxidant defenses (e.g., SOD1), and restore SIRT1/PGC-1α signaling. They suppress the FoxO3a-MuRF1/Atrogin-1 axis and TNF-α/NF-κB inflammatory pathways, ultimately preserving mitochondrial function and muscle integrity (Bellanti et al., 2022). ROS-induced activation of the autophagy-lysosomal pathway (ALP) promotes muscle degradation (see Supplementary Appendix Figure 3 in the attachment for detailed content).

## 6 Mitochondria-targeted therapeutic strategies for ROS regulation

### 6.1 Antioxidant compounds

Antioxidants play a pivotal role in combating skeletal muscle atrophy by neutralizing excessive reactive oxygen species (ROS) or enhancing endogenous defense systems. Vitamins are among the most extensively studied antioxidants. Vitamin C (ascorbic acid), a water-soluble compound, directly scavenges hydroxyl and superoxide radicals, and has been shown to inhibit the expression of Atrogin-1 and MuRF1, thereby reducing ROS-induced proteolysis and delaying muscle wasting in animal models (Takisawa et al., 2019). Vitamin E (α-tocopherol), a lipid-soluble antioxidant, stabilizes cell membranes and protects muscle fibers against oxidative damage, particularly under conditions like hindlimb unloading (Chung et al., 2018; Takisawa et al., 2019). Vitamin D exerts anti-atrophic effects by modulating inflammatory pathways and supporting mitochondrial biogenesis (Chung et al., 2018) (Table 1).

**TABLE 1:** Antioxidant compounds.

Category	Compound	Function	References
Vitamins	Vitamin C	Scavenges hydroxyl and superoxide radicals; inhibits Atrogin-1 and MuRF1 expression	Takisawa et al. (2019)
	Vitamin E	Stabilizes cell membranes; reduces oxidative stress and muscle atrophy	Chung et al. (2018)
	Vitamin D	Anti-inflammatory; regulates mitochondrial function; improves muscle quality	Chung et al. (2018)
Amino acid derivatives	S-allyl cysteine	Inhibits ROS production; protects muscle fiber structure	Gupta et al. (2020)
	Taurine	Antioxidant and anti-inflammatory; relieves damage induced by aging/high glucose	Liu et al. (2022)
Natural small molecules	PQQ (Pyrroloquinoline quinone)	Scavenges ROS; activates PGC-1α; promotes mitochondrial biogenesis	Yt et al. (2015)
Herbal extracts	Silybin	Regulates FoxO pathway; alleviates chemotherapy-induced muscle atrophy	My et al. (2022)
	Isoquercitrin, Morin	Upregulate antioxidant enzymes; inhibit NOX expression	Zhang et al. (2022)
	Curcumin	Inhibits GSK-3β and mitochondrial damage	Qian et al. (2021)
	Paeoniflorin	Activates AMPK/SIRT1/PGC-1α axis; improves mitochondrial function	Anon, (2025)
	Atractylenolide III	Activates antioxidant enzymes and mTOR pathway; suppresses autophagy	Tudorachi et al. (2021)
	Ginsenoside Rb1	Inhibits NF-κB/caspase signaling; protects MuSCs	Shi et al. (2025)
Others	Glutathione (GSH)	Major intracellular antioxidant; maintains redox cycle	Salagre et al. (2023)
	NAC (N-acetylcysteine)	GSH precursor; replenishes stores; boosts antioxidant defense	Lui et al. (2022)

Beyond vitamins, various natural compounds offer antioxidant and anti-atrophic benefits. S-allyl cysteine (from garlic) and taurine (a sulfur-containing amino acid) suppress ROS accumulation and maintain muscle fiber integrity, particularly in denervation- or aging-related atrophy (Gupta et al., 2020). Pyrroloquinoline quinone (PQQ) activates the PGC-1α pathway, restoring mitochondrial function, reducing MuRF1 expression, and alleviating TNF-α–induced muscle atrophy (Yt et al., 2015). Plant polyphenols such as quercetin exhibit strong antioxidative and anti-inflammatory activity by scavenging radicals, chelating metals, and activating the Nrf2–ARE pathway, thereby upregulating antioxidant enzymes like SOD, catalase (CAT), and glutathione peroxidase (GPx). Silybin, isoquercitrin, and morin inhibit pro-oxidant enzymes (e.g., NOX2/NOX4) and enhance endogenous antioxidants, providing protection in various muscle atrophy models (Ryu et al., 2019).

A range of phytochemicals further modulate key catabolic pathways. Curcumin inhibits GSK-3β and restores mitochondrial function in chronic muscle wasting (Zhang et al., 2022). Paeoniflorin activates the AMPK/SIRT1/PGC-1α axis to mitigate mitochondrial dysfunction, while atractylenolide III upregulates antioxidant enzymes and activates the PI3K/Akt/mTOR pathway to inhibit excessive autophagy (b). Ginsenoside Rb1 blocks NF-κB and caspase pathways to preserve muscle stem cell viability (Shi et al., 2025). Additionally, endogenous antioxidants such as glutathione (GSH), melatonin, and N-acetylcysteine (NAC) play essential roles in maintaining redox balance, detoxifying ROS, and protecting mitochondrial integrity. Coenzyme Q10 (CoQ10), a critical component of the electron transport chain, improves mitochondrial respiration and reduces oxidative stress in both skeletal and cardiac muscle (Salagre et al., 2023).

### 6.2 Mitochondria-targeted antioxidants

Targeting mitochondrial-derived reactive oxygen species (mtROS) has emerged as a promising strategy to restore metabolic homeostasis, attenuate proteolysis, and preserve skeletal muscle function (Tudorachi et al., 2021). Several mitochondria-targeted antioxidants have demonstrated notable anti-atrophic effects in preclinical models. MitoQ, a mitochondria-penetrating ubiquinone derivative, restores mitochondrial redox balance and significantly alleviates cachexia-induced muscle wasting in C26 tumor-bearing mice without affecting tumor burden. Mechanistically, MitoQ suppresses the expression of Atrogin-1 and MuRF1, enhances mitochondrial β-oxidation, and improves overall energy metabolism and proteostasis (Pin et al., 2022). Similarly, SkQ1, a plastoquinone-based antioxidant, shows sex-specific protective effects-enhancing protein synthesis and muscle mass in males, while improving calcium homeostasis and contractility in females. It also reduces fatigue in early-stage ovarian cancer cachexia, suggesting potential for early therapeutic intervention (Tsitkanou et al., 2024).

SS-31 (elamipretide), a mitochondria-targeting tetrapeptide that binds cardiolipin, improves mitochondrial structure and bioenergetics, and has shown efficacy in clinical trials for heart failure, ischemia-reperfusion injury, and mitochondrial myopathies (Campbell et al., 2019). EUK-134, a synthetic mimetic with both superoxide dismutase (SOD) and catalase activities, scavenges cytosolic ROS and prevents nNOS mislocalization and NOX2 overactivation in disuse- and aging-induced atrophy. It also downregulates proteolytic gene expression and enhances antioxidant responses, contributing to sarcolemmal stability and muscle preservation under unloading conditions (Kamal and Trombetta-Lima, 2025).

It should be noted that the current evidence supporting mtROS-targeted interventions is predominantly derived from cellular and animal models, with relatively limited validation in rotator cuff-specific injury models and clinical settings. Key translational challenges include optimizing dosing strategies to balance physiological and pathological ROS signaling, identifying appropriate therapeutic windows during the injury–repair continuum, and achieving effective tissue-specific delivery to the supraspinatus muscle. Moreover, differences between generalized disuse models and the unique biomechanical and ischemic environment of rotator cuff injury may limit direct clinical extrapolation.

## 7 Conclusion

Supraspinatus muscle atrophy secondary to rotator cuff injury represents a progressive and frequently irreversible degenerative process that profoundly compromises shoulder function and limits postoperative recovery. Accumulating evidence highlights that mitochondrial reactive oxygen species (ROS) imbalance is not merely a byproduct of tissue damage but acts as a central pathological driver, initiating and sustaining skeletal muscle wasting. This review has delineated the anatomical and pathophysiological basis of rotator cuff-associated muscle atrophy, with a particular focus on the pivotal role of ROS in mediating oxidative damage, mitochondrial dysfunction, myofiber apoptosis, and activation of key catabolic signaling cascades, including the FOXO, NF-κB, and MAPK pathways. Furthermore, it examined how ROS dysregulation modulates the ubiquitin-proteasome system (UPS) and the autophagy-lysosomal pathway (ALP), exacerbating proteolytic degradation. In addition, this review provided an overview of current therapeutic strategies targeting ROS, including both mitochondria-targeted antioxidants and conventional agents. These interventions show promising potential to attenuate oxidative stress, preserve mitochondrial integrity, and inhibit protein catabolism, thereby offering a solid theoretical foundation for the development of novel treatment approaches aimed at preventing or reversing supraspinatus muscle atrophy.
