# Supplementary material for: Mitochondrial ROS dyshomeostasis: a key driver of accelerated supraspinatus atrophy after rotator cuff injury
Source: Front Physiol. 2026 Mar 12;17:1783596. doi: 10.3389/fphys.2026.1783596 (PMC13017390; doi:10.3389/fphys.2026.1783596)
Supplement: Supplementary file 1 [file Supplementaryfile1.docx]

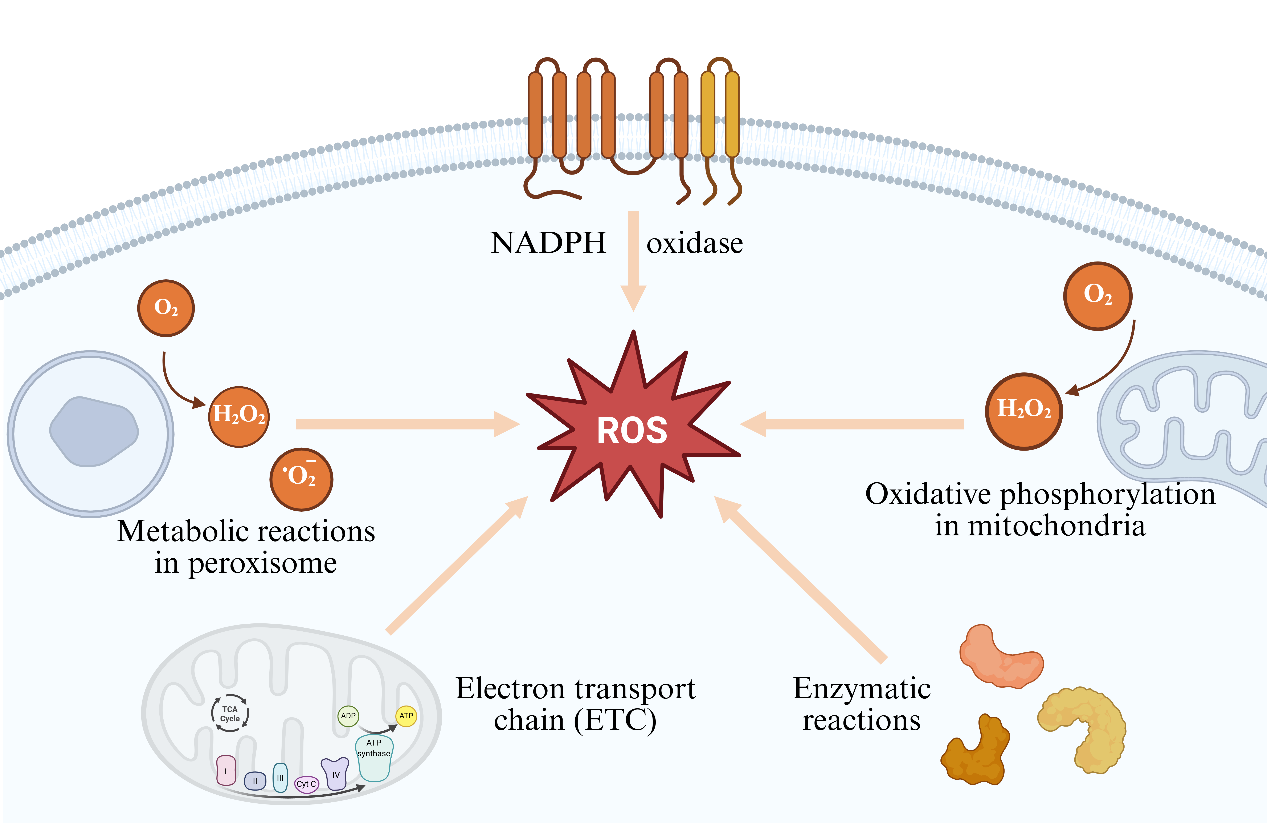


Appendix Figure 1. Major intracellular sources of reactive oxygen species (ROS)

This figure highlights key sources of ROS within cells: (1) the mitochondrial electron transport chain (ETC), where electron leakage during oxidative phosphorylation produces superoxide; (2) peroxisomal metabolism, including fatty acid and amino acid oxidation; (3) NADPH oxidases (NOX), which generate ROS by transferring electrons to oxygen; and (4) other oxidase enzymes, such as xanthine oxidase and cyclooxygenases.


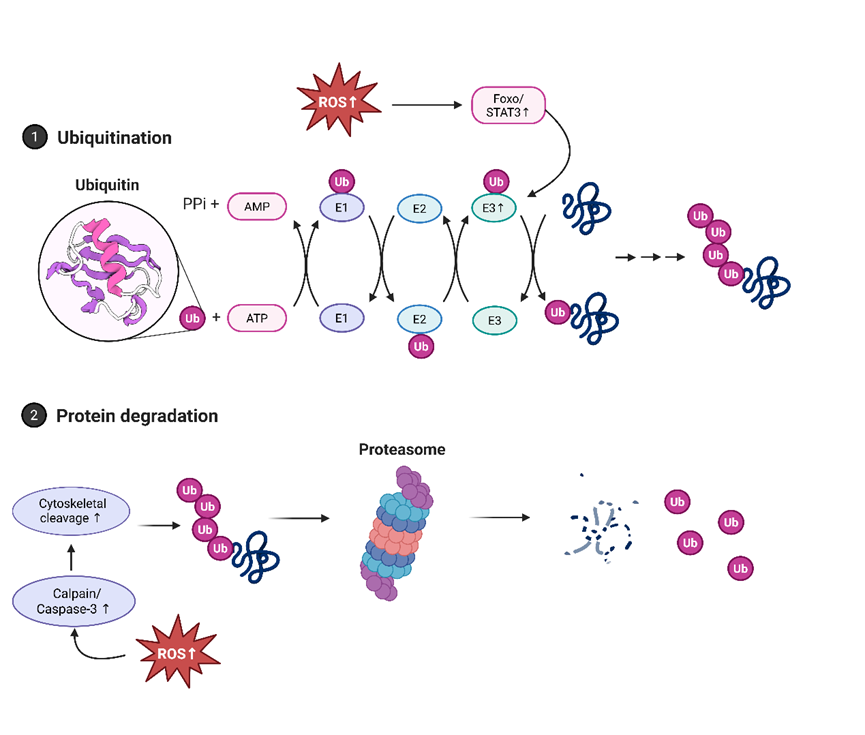


Appendix Figure 2. ROS accelerates skeletal muscle protein degradation via activation of the UPS

Elevated ROS levels activate FoxO and STAT3, enhancing transcription of E3 ligases Atrogin-1 and MuRF1, which mediate protein ubiquitination. Concurrently, ROS-induced caspase-3 and calpain activity fragments structural proteins into UPS substrates. These are then degraded by the 26S proteasome, driving sustained muscle proteolysis.


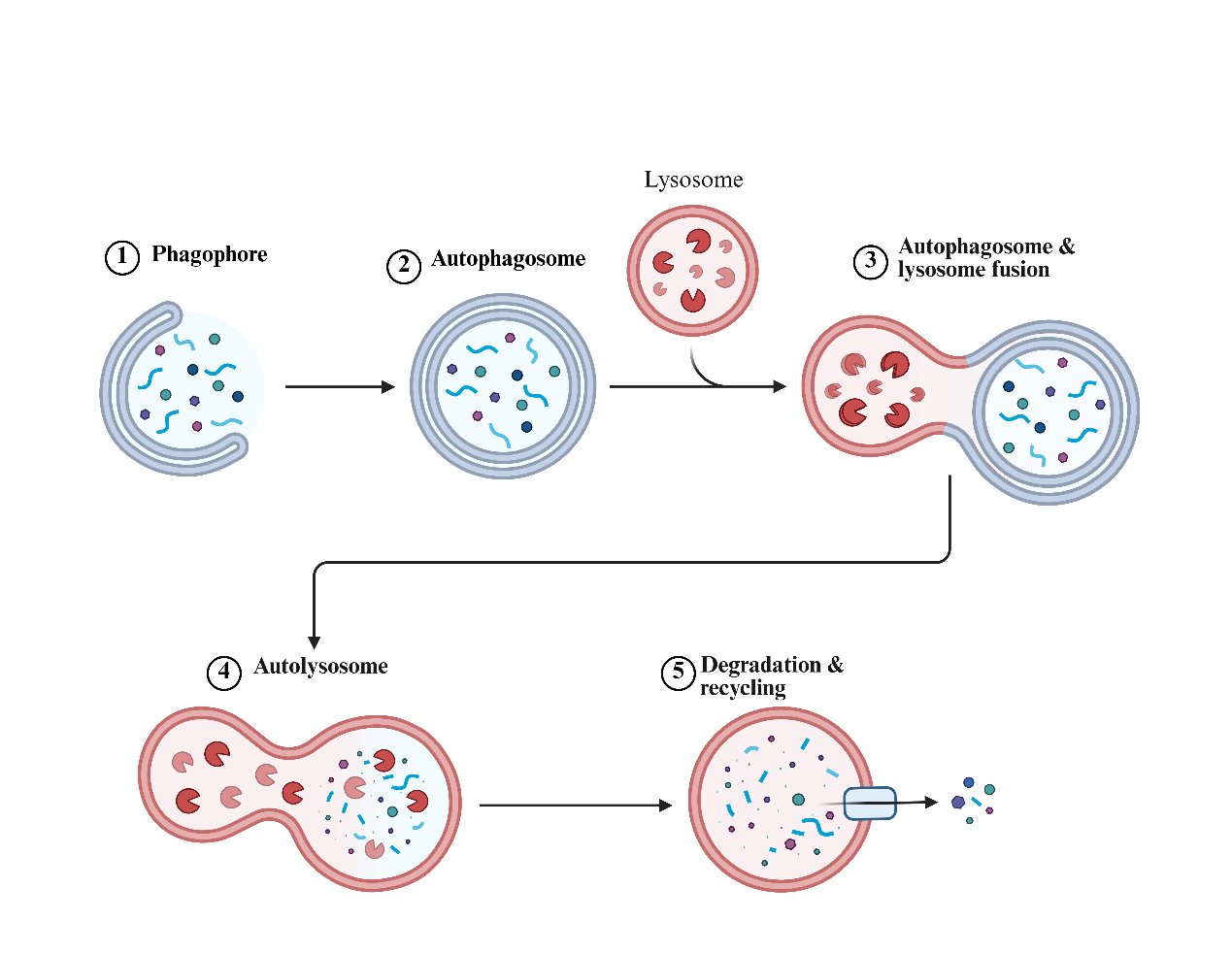


Appendix Figure 3. ROS-induced activation of the autophagy–lysosomal pathway (ALP) promotes muscle degradation

The ALP proceeds through five steps: phagophore initiation, autophagosome maturation, lysosome fusion, autolysosome formation, and content degradation. ROS elevation activates TFEB, promoting lysosomal biogenesis and autophagic flux. Excessive activation leads to degradation of mitochondria and contractile proteins, contributing to muscle atrophy.
